# Renal Outcomes of Pioglitazone Compared with Acarbose in Diabetic Patients: A Randomized Controlled Study

**DOI:** 10.1371/journal.pone.0165750

**Published:** 2016-11-03

**Authors:** Yu-Hsin Chen, Der-Cherng Tarng, Harn-Shen Chen

**Affiliations:** 1 Division of Nephrology, Department of Medicine, Taipei Veterans General Hospital, Taipei, Taiwan; 2 Division of Endocrinology and Metabolism, Department of Medicine, Taipei Veterans General Hospital, Taipei, Taiwan; 3 Faculty of Medicine, National Yang-Ming University, Taipei, Taiwan; 4 Department and Institute of Physiology and Institute of Clinical Medicine, National Yang-Ming University, Taipei, Taiwan; Weill Cornell Medical College Qatar, QATAR

## Abstract

**Objective:**

To assess the effect of pioglitazone on renal outcome, including urinary albumin excretion and estimated glomerular filtration rate (eGFR), in diabetic patients.

**Design:**

A prospective, randomized, open-labeled, controlled study.

**Setting:**

Taipei Veterans General Hospital.

**Patients:**

Sixty type 2 diabetic patients treated with sulfonylureas and metformin, whose glycated hemoglobin (HbA1c) levels were between 7% and 10% and eGFR was between 45 and 125 mL/min/1.73 m^2^.

**Intervention:**

The patients were randomized to receive acarbose or pioglitazone and followed up for 6 months. Thirty patients were randomly assigned to receive acarbose, and 30 patients were assigned to receive pioglitazone.

**Measurements:**

The primary study endpoint was the changes in the urinary albumin-to-creatinine ratio (UACR). The secondary endpoint was the changes in eGFR and other parameters.

**Results:**

After 6 months of treatment, the mean changes in UACR were −18 ± 104 and 12 ± 85 (*p* = 0.25, between groups) for the acarbose and pioglitazone groups, respectively. The mean changes in eGFR were 0 ± 14 and −7 ± 16 mL/min/1.73 m^2^ (*p* = 0.09, between groups) for the acarbose and pioglitazone groups, respectively. The reductions in HbA1c were similar in both groups. Fasting blood glucose was lower in the pioglitazone group than in the acarbose group. Significant body weight gain was observed in the pioglitazone group as compared with the acarbose group (1.3 ± 2.8 vs. −0.6 ± 1.5 kg, *p* = 0.002).

**Conclusion:**

In type 2 diabetic patients who were treated with sulfonylureas and metformin and possessed HbA1c levels between 7% and 10%, additional acarbose or pioglitazone for 6 months provided similar glycemic control and eGFR and UACR changes. In the pioglitazone group, the patients exhibited significant body weight gain.

**Trial Registration:**

ClinicalTrials.gov NCT01175486

## Introduction

Diabetic end-stage renal disease increases the risk of cardiovascular events and mortality [1,2]. It is important to prevent or delay progression of diabetic nephropathy in order to reduce the subsequent morbidity and mortality. Thiazolidinediones (TZDs) are one kind of oral anti-diabetic drugs. TZDs are insulin sensitizers that decrease plasma glucose and improve the lipid profile of type 2 diabetic patients [3]. TZDs initiate their action by binding to peroxisome proliferator-activated receptor-γ (PPARγ) [3]. Although PPARγ receptors are most abundant in fat cells [3,4], they have also been demonstrated in the mesangium and glomerulus of kidney [5,6]. Studies on rodents have suggested that TZDs can prevent diabetic nephropathy [6–8]. TZDs reduce urinary albumin excretion and proteinuria in diabetic nephropathy [9,10]. TZDs may control blood glucose as well as protect kidney. Till now, studies investigating the impact of TZDs on the estimated glomerular filtration rate (eGFR) are few, and the results are conflicting. Therefore, we conducted a randomized controlled study to investigate the effects of pioglitazone, a kind of TZD, on urinary albumin excretion and eGFR in type 2 diabetic patients as compared with those treated with acarbose.

## Materials and Methods

### Study design

This prospective, randomized, open-labeled, controlled study was conducted in Taipei Veterans General Hospital to assess the effects of TZDs on diabetic nephropathy. We enrolled type 2 diabetic patients who had been treated with sulfonylureas and metformin, and their glycated hemoglobin (HbA1c) levels were between 7% and 10%. These patients were randomly assigned to additional 50 mg of acarbose treatment three times per day or 30 mg of pioglitazone once per day for a 6-month intervention period. These patients were followed up for 6 months to investigate the short-term effects on diabetic nephropathy. This study was approved by the Institutional Review Board of Taipei Veterans General Hospital, and all clinical investigation was conducted according to the principles expressed in the Declaration of Helsinki. Written informed consent was given before the randomization.

### Subjects

Type 2 diabetic patients who were regularly followed up in Taipei Veterans General Hospital were invited to participate in this study. Inclusion criteria were type 2 diabetes, between 20 and 80 years old, receiving sulfonylureas and metformin treatment, and HbA1c between 7% and 10%. The exclusion criteria were insulin treatment and/or eGFR <45 or >125 mL/min/1.73 m^2^. Patients with cardiovascular diseases, malignancy, pregnancy, acute illness, congestive heart failure (according to the New York Heart Association functional class III to IV), or liver cirrhosis were also excluded.

### Procedures

The baseline information, including age, gender, diabetic duration, body mass index, blood pressure, smoking status, laboratory data, and concomitant medicine, was collected upon enrollment. The body weight, blood pressure, HbA1c concentration, fasting blood glucose, lipid profile, serum creatinine concentration, and urinary albumin-to-creatinine ratio (UACR) were examined at 12 and 24 weeks after randomization. The glomerular filtration rate was estimated using a prediction formula from the four-variable Modification of Diet and Renal Disease study equation [11]. All assessments of urine and blood were performed at a central laboratory.

Patients continued to receive their usual care according to our national guideline for diabetes. Arterial hypertension was treated via a stepwise approach with the target blood pressure <140/90 mmHg. Dyslipidemia was treated with statins to reach the targets of serum total cholesterol <160 mg/dL. No restriction on dietary salt or protein was implemented.

### Assays

HbA1c was measured by high-performance liquid chromatography (HLC-723G7, Tosoh, Japan) with a reference range of 4.2%–5.8%. The inter-assay coefficients of variance were <2% at mean HbA1c levels between 4.4% and 8.2%. The urinary albumin concentration was determined by nephelometry, whereas the serum creatinine concentration was examined by Jaffe reaction with a Hoffmann–LaRoche kit. The glomerular filtration rate was estimated using a prediction formula from the four-variable Modification of Diet and Renal Disease study equation [11].

### Outcome measures

The primary study outcome was the changes in UACR. The secondary outcome was the alteration in eGFR. Other outcomes were the variation in glycemic control and body weight.

### Statistical analysis

Measurements were summarized using the mean ± standard deviation or using the median with interquartile range (IQR) as appropriate. Comparisons between pioglitazone and acarbose groups were performed using the two sample *t*-test for those variates that were approximately normally distributed and the Wilcoxon-Mann-Whitney test otherwise. Categorical data were summarized using percentages and group comparisons were performed using the χ^2^ test. Comparisons between pre and post treatment within each group were performed using the paired *t*-test. Comparisons of changes from pre to post treatment between groups were performed using the two sample *t*-test. Statistical significance was declared at the 0.05 level. Statistical analysis was performed with SPSS for Windows version 18.0 (SPSS, Inc., Chicago, IL, USA).

## Results

**Fig 1:**
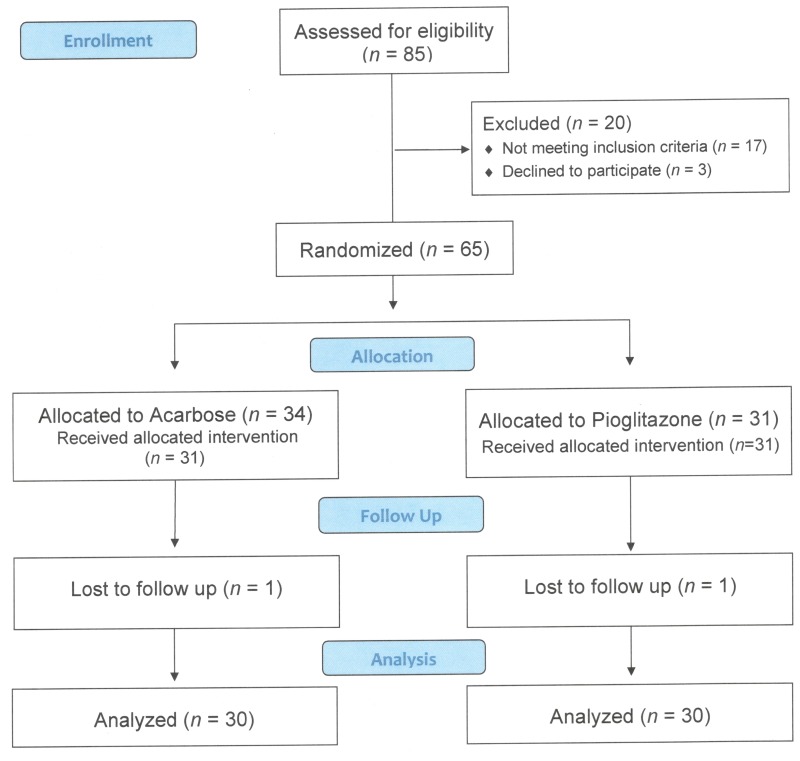
Flow diagram of the study.

We screened 85 type 2 diabetic subjects treated with sulfonylureas and metformin between July 2010 and June 2012 after two years of recruitment. In total, 65 patients were randomly assigned to additional acarbose (*n* = 34) or pioglitazone (*n* = 31) treatment, and 60 patients completed the 6 months of intervention (Fig 1). Baseline characteristics were generally similar between two groups, except there were more women, fewer smokers, and less use of angiotensin-converting enzyme (ACE) inhibitors or angiotensin II receptor blockers (ARBs) in the pioglitazone group (Table 1). The mean age of the participants was 66.7 years, mean diabetes duration was 11.9 years, mean body weight was 67.4 kg, mean body mass index was 28.1 kg/m^2^, mean systolic blood pressure was 135 mmHg, mean fasting blood glucose was 171 mg/dL, mean HbA1c was 8.26%, mean serum creatinine concentration was 0.84 mg/dL, mean eGFR was 88 mL/min/1.73 m^2^, and median UACR was 18 mg/g.

**Table 1:** Baseline characteristics of the study patients.

	Acarbose	Pioglitazone	*p*
	*n* = 30	*n* = 30	
Age (years)	67.2 ± 7.6	66.3 ± 8.8	0.67
Gender-female (%)	36.7	73.3	0.004
Duration of diabetes (years)	12.3 ± 7.4	11.6 ± 4.0	0.67
Body weight (kg)	69.7 ± 10.1	65.1 ± 11.4	0.10
Body mass index	30.1 ± 18.4	26.0 ± 3.4	0.26
Systolic BP (mmHg)	134 ± 16	136 ± 14	0.69
Diastolic BP (mmHg)	79 ± 10	78 ± 10	0.84
Smoking (%)	23.3	3.6	0.03
Laboratory data			
HbA1c (%)	8.2 ± 0.8	8.3 ± 0.8	0.88
Fasting blood glucose (mg/dL)	167 ± 33	176 ± 43	0.35
Total cholesterol (mg/dL)	181 ± 26	177 ± 40	0.64
HDL cholesterol (mg/dL)	47 ± 10	49 ± 21	0.72
LDL cholesterol (mg/dL)	106 ± 21	101 ± 33	0.52
Triglycerides (mg/dL)	141 ± 58	143 ± 74	0.88
ALT (IU/L)	30 ± 16	25 ± 12	0.20
Creatinine (mg/dL)	0.90 ± 0.24	0.79 ± 0.25	0.07
eGFR (mL/min/1.73 m^2^)	85 ± 20	91 ± 24	0.31
UACR (median, IQR) (mg/g)	18 [7–66]	8 [5–54]	0.36
Medicine			
Alpha-blockers (%)	6.7	6.7	1.00
Beta-blockers (%)	33.3	26.7	0.57
Calcium channel blockers (%)	33.3	26.7	0.57
Diuretics (%)	16.7	6.7	0.23
ACE inhibitors/ARBs (%)	60.0	26.7	0.009
Other anti-hypertensive agents (%)	6.7	6.7	1.00
Fibrate (%)	0	0	NA
Statin (%)	43.3	53.3	0.44
Aspirin (%)	20	20	1.00

Continuous data are expressed as the mean ± standard deviation and the comparisons are performed using the two sample *t*-test. UACR are expressed as median with interquartile range and are compared using Wilcoxon-Mann-Whitney test. Categorical data are expressed in percentages and group comparisons are performed using the χ^2^ test.

Abbreviations: ACE, angiotensin-converting enzyme; ALT, alanine aminotransferase; ARBs, angiotensin II receptor blockers; BP, blood pressure; eGFR, estimated glomerular filtration rate; IQR, interquartile range; UACR, urinary albumin-to-creatinine ratio.

After 6 months of intervention, HbA1c and fasting plasma glucose improved in both groups. The reductions in HbA1c were similar in both groups. Fasting blood glucose was lower in the pioglitazone group than in the acarbose group. Significant body weight gain was observed in the pioglitazone group as compared with the acarbose group (1.3 ± 2.8 vs. −0.6 ± 1.5 kg, p = 0.002). After 6 months of treatment, The mean changes in UACR were −18 ± 104 and 12 ± 85 (p = 0.25, between groups) for the acarbose and pioglitazone groups, respectively. The mean changes in eGFR were 0 ± 14 and −7 ± 16 mL/min/1.73 m2 (p = 0.09, between groups) for the acarbose and pioglitazone groups, respectively.

The within-group changes before and after intervention are shown in Table 2. Blood pressures and lipid profile were similar before and after treatment, except for a significant reduction in the triglyceride level in the acarbose group. Table 3 illustrates that no significant differences in body weight, HbA1c level, eGFR, and UACR between two groups. The patients in the pioglitazone group had more body weight gain and more reduction in fasting blood glucose than those in the acarbose group.

**Table 2:** Within-group changes before and after 6 months of intervention.

	Acarbose (*n* = 30)			Pioglitazone (n = 30)		
	Change	95% CI	*p*	Change	95% CI	*p*
Body weight (kg)	-0.6 ± 1.5	-1.17 to -0.06	0.03	1.3 ± 2.8	0.25 to 2.34	0.02
Systolic BP (mmHg)	-1 ± 15	-6.68 to 4.74	0.73	-2 ± 14	-7.15 to 3.35	0.47
Diastolic BP (mmHg)	-3 ± 11	-6.85 to 1.25	0.17	-1 ± 9	-4.81 to 1.95	0.39
HbA1c (%)	-0.5 ± 0.9	-0.76 to -0.13	0.007	-0.5 ± 0.6	-0.72 to -0.28	<0.001
Fasting blood glucose (mg/dL)	-6 ± 32	-18.26 to 6.05	0.31	-28 ± 41	-42.80 to -12.34	0.001
Total cholesterol (mg/dL)	-6 ± 29	-16.50 to 5.50	0.32	-5 ± 44	-21.31 to 11.64	0.55
HDL cholesterol (mg/dL)	1 ± 9	-2.13 to 5.03	0.41	3 ± 9	-0.34 to 6.68	0.08
LDL cholesterol (mg/dL)	-3 ± 23	-11.47 to 6.07	0.53	-4 ± 41	-19.86 to 11.09	0.57
Triglycerides (mg/dL)	-23 ± 54	-42.90 to -2.24	0.03	-14 ± 42	-30.05 to 1.25	0.07
Creatinine (mg/dL)	0 ± 0.13	-0.06 to 0.04	0.80	0.05 ± 0.14	-0.001 to 0.10	0.06
eGFR (mL/min/1.73 m^2^)	0 ± 14	-5.19 to 5.39	0.97	-7 ± 16	-12.88 to -0.68	0.03
UACR (mg/g)	-18 ± 104	-58.16 to 21.29	0.35	12 ± 85	-20.46 to 44.06	0.46

Data are expressed as the mean ± SD. The comparisons are performed using the paired *t*-test.

Abbreviations: BP, blood pressure; CI, confidence of interval; eGFR, estimated glomerular filtration rate; UACR, urinary albumin-to-creatinine ratio.

**Table 3:** Between-group differences of outcomes after 6 months of intervention.

	Acarbose	Pioglitazone	95% CI	*p*
Body weight (Kg)	-0.6 ± 1.5	1.3 ± 2.8	-3.07 to -0.76	0.002
HbA1c (%)	-0.5 ± 0.9	-0.5 ± 0.6	-0.32 to 0.42	0.78
Fasting blood glucose (mg/dL)	-6 ± 32	-28 ± 41	2.32 to 40.62	0.03
eGFR (mL/min/1.73 m^2^)	0 ± 14	-7 ± 16	-1.04 to 14.77	0.09
UACR (mg/g)	-18 ± 104	12 ± 85	-74.32 to 19.91	0.25

Data are expressed as the mean ± SD. The comparisons are performed using the two sample *t*-test.

Abbreviations: CI, confidence of interval; eGFR, estimated glomerular filtration rate; UACR, urinary albumin-to-creatinine ratio.

The adverse events were recorded. No severe hypoglycemia occurred in either group. The overall rates of minor to moderate hypoglycemia showed no significant difference between two groups (Acarbose vs. Pioglitazone: 1.37 ± 1.16 vs. 1.87 ± 1.48 episodes, *p* = 0.51). The most common side-effect was mild to moderate gastrointestinal symptoms in those given acarbose than in those given pioglitazone (Acarbose vs. Pioglitazone: 83% vs. 60%, *p* <0.001). However in the pioglitazone group, more patients had edema (Acarbose vs. Pioglitazone: 3.3% vs. 36.7%, *p* <0.001) and body weight increase (Acarbose vs. Pioglitazone: −0.6 ± 1.5 kg vs. 1.3 ± 2.8 kg, *p* = 0.002).

## Discussion

This clinical study compared the renal outcomes and glycemic control in diabetic patients who had taken metformin and sulfonylureas via additional pioglitazone or acarbose treatment for 6 months. The results showed similar glycemic control in two groups, and no significant differences of changes in eGFR and UACR between pioglitazone and acarbose treatment. Mean body weight gain was significant in the pioglitazone group. This is the first randomized controlled study using UACR and eGFR as the study outcomes.

The strength of this randomized controlled study is that the baseline HbA1c levels (Acarbose vs. Pioglitazone: 8.2 ± 0.8% vs. 8.3 ± 0.8%, *p* = 0.88) and HbA1c changes (Acarbose vs. Pioglitazone: -0.5 ± 0.9% vs. -0.5 ± 0.6%, *p* = 0.78) were the same between the two groups. The acarbose group had a similar glycemic control with pioglitazone group before and after intervention. We chose acarbose as a control to make glycemic control comparable. Only one animal study showed that acarbose, an alpha-glucosidase inhibitor, reduced increased albumin excretion in streptozotocin-diabetic rats [12]. No human study has demonstrated the influence of acarbose on renal function. If we used placebo as a control, a different glycemic control might affect the renal outcome.

TZDs reduce urinary albumin excretion and proteinuria in diabetic nephropathy [9,10]. Inhibition of transforming growth factor-β 1 and genes involved in collagen/fibronectin formation and reduced serum/renal interstitial tumor necrosis factor-α levels [13,14] have been suggested as potential mechanisms for the renoprotective effect of TZDs [6], but the precise mechanism by which TZDs reduce albuminuria remains unknown. In this study, pioglitazone use for 6 months increased UACR to 12 ± 85 mg/g. As compared with the acarbose group, the change was not significant.

The influence of TZDs on eGFR is not clear. A retrospective study showed that among diabetic patients with normal renal function, rosiglitazone users exhibited a lower decline in renal function than controls during a 3-year follow-up period [15]. Our previous nationwide cohort study showed that TZD users were associated with a low risk for long-term dialysis [16]. However, the hard endpoint of our previous study, long-term dialysis, might reflect not only the decline in renal function but also the cardiovascular effects of TZDs in patients with advanced chronic kidney disease. Rosiglitazone treatment caused a decline of renal function in diabetic patients as compared with control group over 5 years of follow-up in a retrospective cohort study [17]. In the PROactive study, which enrolled diabetic patients with documented macrovascular disease, the *post hoc* analysis showed pioglitazone group had a greater decline of eGFR as compared with the control group [18]. A randomized controlled trial showed a 5.4% decrease in eGFR after 52 weeks of pioglitazone treatment in stage 3 chronic kidney disease with type 2 diabetes mellitus [19]. They found that the decline in eGFR plateaued after 8 weeks of treatment and improved to a 3.4% decrease from baseline eGFR after 8 weeks of treatment [19]. In our study, the mean eGFR had a non-significant decrement (95% CI -1.04 to 14.77, *p* = 0.09) in the pioglitazone group compared with the acarbose group. To date, the mechanism for eGFR reduction with PPARγ agonists is not well characterized.

In this study, the patients receiving pioglitazone had significant body weight gain (1.3 ± 2.8 kg) during 6 months of treatment compared with those in the acarbose group (−0.6 ± 1.5 kg). This finding is unsurprising because fluid retention is a known side effect of TZDs. Fluid retention and peripheral edema are described in 5%–7% of patients using TZDs alone or in conjunction with other oral agents or up to 15% of patients using pioglitazone with insulin [20]. TZDs can cause fluid retention and peripheral edema in diabetic patients [20–22].

The limitations of this study were a small sample size, short follow-up period, and minimal differences at baseline between two drugs, such as percentage of female patients and smoking status. There were 60.0% and 26.7% of patients in the acarbose and pioglitazone groups receiving ACE inhibitors or ARBs at baseline. Since ACE inhibitors or ARBs may have ameliorated a greater increase in urinary albumin excretion, the decreased urinary albumin-to-creatinine ratio after treatment might partially reflect the increased use of ACE inhibitors or ARBs in the acarbose group.

In conclusion, in patients with type 2 diabetes who were treated with sulfonylureas and metformin and demonstrated HbA1c between 7% and 10%, additional acarbose or pioglitazone treatment for 6 months could improve glycemic control and exert a similar renal effect on eGFR and UACR. However, the patients receiving pioglitazone exhibited more body weight gain than those receiving acarbose. Given the small sample size and short follow-up period in this study, large size studies with longer follow-up period are needed in the future.

## Supporting Information

S1 File Protocol. (PDF)

S2 File IRB for Thiazolidinedione. (PDF)

S3 File CONSORT 2010 checklist. (DOC)

## References

[1] Chang YT, Wu JL, Hsu CC, Wang JD, Sung JM. Diabetes and end-stage renal disease synergistically contribute to increased incidence of cardiovascular events: a nationwide follow-up study during 1998–2009. Diabetes care 2014;37:277–285. 10.2337/dc13-0781 23920086

[2] Wang AY. Cardiovascular risk in diabetic end-stage renal disease patients. J Diabetes 2011;3:119–131. 10.1111/j.1753-0407.2011.00113.x 21599866

[3] Yki-Jarvinen H. Thiazolidinediones. N Engl J Med 2004;351:1106–1118. 10.1056/NEJMra041001 15356308

[4] Bays H, Mandarino L, DeFronzo RA. Role of the adipocytes FFA and ectopic fat in the pathogenesis of type 2 diabetes mellitus. PPAR agonists provide a rational therapeutic approach. J Clin Endocrinol Metab 2004;89:463–478. 10.1210/jc.2003-030723 14764748

[5] Asano T, Wakisaka M, Yoshinari M, Iino K, Sonoki K, Iwase M et al Peroxisome proliferator-activated receptor gamma1 (PPARgamma1) expresses in rat mesangial cells and PPARgamma agonists modulate its differentiation. Biochim Biophys Acta 2000;1497:148–154. 10838168 10.1016/s0167-4889(00)00054-9

[6] Guan Y, Breyer M. Peroxisome proliferator-activated receptors (PPARs). Novel therapeutic targets in renal disease. Kidney Int 2001;60:14–30. 10.1046/j.1523-1755.2001.00766.x 11422732

[7] Isshiki K, Haneda M, Koya D, Maeda S, Sugimoto T, Kikkawa R. Thiazolidinedione compounds ameliorate glomerular dysfunction independent of their insulinsensitizing action in diabetic rats. Diabetes 2000;49:1022–1032. 10866056 10.2337/diabetes.49.6.1022

[8] McCarthy KJ, Routh RE, Shaw W, Walsh K, Welbourne TC, Johnson JH. Troglitazone halts diabetic glomerulosclerosis by blockade of mesangial expansion. Kidney Int 2000;58:2341–2350. 10.1046/j.1523-1755.2000.00418.x 11115068

[9] Sarafidis PA, Bakris GL. Protection of the kidney by thiazolidinediones: an assessment from bench to bedside. Kidney Int 2006;70:1223–1233. 10.1038/sj.ki.5001620 16883325

[10] Sarafidis PA, Stafylas PC, Georgianos PI, Saratzis AN, Lasaridis AN. Effect of thiazolidinediones on albuminuria and proteinuria in diabetes: a meta-analysis. Am J Kidney Dis 2010;55:835–847. 10.1053/j.ajkd.2009.11.013 20110146

[11] Stevens LA, Coresh J, Greene T, Levey AS. Assessing kidney function—measured and estimated glomerular filtration rate. N Engl J Med 2006;354:2473–2483. 10.1056/NEJMra054415 16760447

[12] Cohen MP, Vasselli JR, Neuman RG, Witt J. Treatment with acarbose, an alpha-glucosidase inhibitor, reduces increased albumin excretion in streptozotocin-diabetic rats. Gen Pharmacol 1995;26:1355–1361. 7590131 10.1016/0306-3623(94)00283-s

[13] Moriwaki Y, Yamamoto T, Shibutani Y, Aoki E, Tsutsumi Z, Takahashi S et al Elevated levels of interleukin-18 and tumor necrosis factor-a in serum of patients with type 2 diabetes mellitus: relationship with diabetic nephropathy. Metabolism 2003;52:605–608. 10.1053/meta.2003.50096 12759891

[14] Kalantarinia K, Awad AS, Siragy H. Urinary and renal interstitial concentrations of TNF-a increase prior to albuminuria in diabetic rats. Kidney Int 2003;64:1208–1213. 10.1046/j.1523-1755.2003.00237.x 12969138

[15] Kim MK, Ko SH, Baek KH, Ahn YB, Yoon KH, Kang MI et al Long-term effects of rosiglitazone on the progressive decline in renal function in patients with type 2 diabetes. Korean J Intern Med 2009;24:227–232. 10.3904/kjim.2009.24.3.227 19721859 PMC2732782

[16] Chen YH, Chiang MH, Liu JS, Chang YK, Kuo KL, Hung SC et al Thiazolidinediones and risk of long-term dialysis in diabetic patients with advanced chronic kidney disease: a nationwide cohort study. PLoS One 2015;10:e0129922 10.1371/journal.pone.0129922 26083376 PMC4470911

[17] Feldman L, Shani M, Efrati S, Beberashvili I, Baevsky T, Weissgarten J et al Association between rosiglitazone use and decline in renal function in patients with type 2 diabetes mellitus. J Nephrol 2010;23:350–356. 20155725

[18] Schneider CA, Ferrannini E, Defronzo R, Schernthaner G, Yates J, Erdmann E. Effect of pioglitazone on cardiovascular outcome in diabetes and chronic kidney disease. J Am Soc Nephrol 2008;19:182–187. 10.1681/ASN.2007060678 18057215 PMC2391042

[19] Ruilope L, Hanefeld M, Lincoff AM, Viberti G, Meyer-Reigner S, Mudie N et al Effects of the dual peroxisome proliferator-activated receptor-α/γ agonist aleglitazar on renal function in patients with stage 3 chronic kidney disease and type 2 diabetes: a Phase IIb, randomized study. BMC Nephrol 2014;15:180 10.1186/1471-2369-15-180 25407798 PMC4364102

[20] Niemeyer NV, Janney LM. Thiazolidinedione-induced edema. Pharmacotherapy 2002;22:924–929. 12126225 10.1592/phco.22.11.924.33626

[21] Bresnick GH. Diabetic macular edema. A review. Ophthalmology 1986;93:989–997. 3531959 10.1016/s0161-6420(86)33650-9

[22] Mudaliar S, Chang AR, Henry RR. Thiazolidinediones, peripheral edema, and type 2 diabetes: incidence, pathophysiology, and clinical implications. Endocr Pract 2003;9:406–416. 10.4158/EP.9.5.406 14583425

