# Supplementary material for: Renal Outcomes of Pioglitazone Compared with Acarbose in Diabetic Patients: A Randomized Controlled Study
Source: PLoS One. 2016 Nov 3;11(11):e0165750. doi: 10.1371/journal.pone.0165750 (PMC5094682; doi:10.1371/journal.pone.0165750)
Supplement: S1 File — (PDF) [file pone.0165750.s001.pdf]

# 台 北 榮 總 研究計畫申請書

一、基本資料：

申請條碼：100DHA0100181 *100DHA0100181*

| | | | | | |
|---|---|---|---|---|---|
| 計畫類別(單選) | C(資深型計畫) | | | | |
| 研究型別 | 個別型計畫 | | | | |
| 計畫歸屬 | 醫學類 | | | | |
| 申請機構/系所（單位） | 臺北榮民總醫院內科部新陳代謝科 | | | | |
| 本計畫主持人姓名 | 陳涵栩 | 職稱 | 主治醫師 | 身分證號碼 | *******982 |
| 本計畫名稱 中文 | Thiazolidinedione 和糖尿病視網膜病變及腎病變的相關性研究 | | | | |
| 本計畫名稱 英文 | The effects of thiazolidinedione on the diabetic retinopathy and nephropathy | | | | |
| 整合型總計畫名稱 | | | | | |
| 整合型總計畫主持人 | | | | 身分證號碼 | |
| 全程執行期限 | 自民國 100 年 01 月 01 日起至民國 100 年 12 月 31 日 | | | | |
| 研究學門(請參考本申請書所附之學門專長分類表填寫) | 學門代碼 | 名稱(如為其他類，請自行填寫學門) | | | |
| | BV | 腎臟科、新陳代謝及內分泌 | | | |
| 研究性質 | 臨床醫學 | | | | |
| 本計畫是否有進行下列實驗：(勾選下列任一項，須附相關實驗之同意文件)<br>人體實驗 | | | | | |
| 計畫連絡人 | 姓名：陳涵栩　電話：(公) 02-28757515　(宅/手機) 0920734968 | | | | |
| 通訊地址 | 台北市石牌路二段 201 號 台北榮總 內分泌暨新陳代謝科 | | | | |
| 傳真號碼 | 02-28745674 | E-MAIL： | chenhs@vghtpe.gov.tw | | |

計畫主持人(申請人)簽章：________________　　日期：________________

## 二、申請補助經費：

金額單位：新台幣元

| 執行年次<br>補助項目 | | 第一年<br>(2011 年 1 月<br>~2011 年 12 月) | 第二年 | 第三年 | 第四年 | 第五年 |
|---|---|---|---|---|---|---|
| 研究人力費 | | 453,222 | | | | |
| 研究設備費 | | 0 | | | | |
| 研究業務費用 | | 340,000 | | | | |
| 差旅費 | | 0 | | | | |
| | | | | | | |
| | | | | | | |
| | | | | | | |
| 合 計 | | 793,222 | | | | |
| | | | | | | |
| 博士後研究 | 國內、外地區 | 共 0 名 | 共名 | 共名 | 共名 | 共名 |
| | 大陸地區 | 共 0 名 | 共名 | 共名 | 共名 | 共名 |

申請機構或其他單位（含產業界）提供之配合項目（無配合補助項目者免填）

| 配 合 單 位 名 稱 | 配合補助項目 | 配合補助金額 | 配 合 年 次 |
|---|---|---|---|
| | | | |
| | | | |

配合單位主管簽章（或附相關證明文件）：___________________

## 三、主要研究人力：

（一）請依照「主持人」、「共同主持人」、「協同研究人員」及「博士後研究」等類別之順序分別填寫。

| 類 別 | 姓名 | 服務機構/系所 | 職稱 | 在本研究計畫內擔任之具體工作性質、項目及範圍 | ＊每月平均投入工作時數比率(%) |
|---|---|---|---|---|---|
| 主持人 | 陳涵栩 | 臺北榮民總醫院內科部新陳代謝科 | 主治醫師 | 1. 撰寫研究計畫<br>2. 收錄病人及追蹤病人。<br>3. 身體檢查及實驗室檢查的申請。<br>4. 填寫及通報嚴重不良反應(SAE)<br>5. 整理病患資料及撰寫論文。 | 30 % |
| 協同研究人員 | 郭錦松 | 臺北榮民總醫院內科部新陳代謝科 | 主治醫師 | 1. 收錄病人及追蹤病人。<br>2. 身體檢查及實驗室檢查的申請。<br>3. 填寫及通報嚴重不良反應(SAE)<br>4. 整理病患資料。 | 10 % |

※註：每月平均投入工作時數比率係填寫每人每月平均投入本計畫工作時數佔其每月全部工作時間之比率，以百分比表示（例如：50%即表示該研究人員每月投入本計畫研究工作之時數佔其每月全部工時之百分五十）。

（二）如申請博士後研究，請分年列述博士後研究參與本研究計畫之
1.目的及必備之專長。
2.研究項目。
3.工作份量及其對該計畫之影響程度。
4.工作績效評估準則。
5.若已有人選者，請務必填註人選姓名，並將其個人資料表併同本計畫書送本院。

## 四、研究人力費：

金額單位：新台幣元

第 1 年

| （一）專任助理、講師及助教級兼任助理、臨時工資 | | | | | | |
|---|---|---|---|---|---|---|
| 類別/級別 | 人數 | 姓 名 | 工 作 月 數 | 月支酬金（含勞健保費） | 小計 | 請述明：1.最高學歷 2.曾擔任專題研究計畫專任助理之經歷 3.在本計畫內擔任之具體工作性質、項目及範圍 |
| 三專級專任助理第五年 | 1 | | 12 | 29,100 | 392,850 | 1. 製作同意書，病例紀錄格式等。<br>2. 到門診幫忙收錄病人，篩選排入以及排除條件。<br>3. 影印同意書並備份在不同房間。<br>4. 填寫病例紀錄格式並 key in 資料。 |
| 分擔勞健保費(三專級專任助理第五年) | 1 | | 12 | 3,213 | 38,556 | 三專級專任助理第五年 3213 元 x 12 月 x 1 名 |
| 勞退基金 | 1 | | 12 | 1,818 | 21,816 | 三專級專任助理第五年 30300 元 x 12 月 x 6% x 1 名 |
| 合 計（一） | | | | | 453,222 | |

| （二）博士班研究生、碩士班研究生及大專學生兼任助理 | | | | | |
|---|---|---|---|---|---|
| 級別或姓名 | 人數(1) | 每人每月單元數(2) | 獎助月數(3) | 小計 (4)＝＄2000Ｘ(1)Ｘ(2)Ｘ(3) | 在本研究計畫內擔任之具體工作性質、項目及範圍 |
| | | | | | |
| 合計（二） | | | | | |
| 總計（三）＝合計（一）＋合計（二） | | | | 453,222 | |

## 六、研究業務費用：

第 1 年　　　　　　　　　　　　　　　　　　　　　　　金額單位：新台幣元

| 項 目 名 稱 | 說明 | 單位 | 數量 | 單價 | 金額 | 備註 |
|---|---|---|---|---|---|---|
| 試劑耗材費 | | | 1 | 50,000 | 50,000 | |
| 調查訪視費 | | | 100 | 500 | 50,000 | |
| 維修費 | | | 1 | 20,000 | 20,000 | |
| 文具 | | | 1 | 20,000 | 20,000 | |
| 受試者補助費 | 我們將 Randomize 300 個受試者，一次給五百元。 | 件 | 300 | 500 | 150,000 | |
| 電腦週邊耗材費 | | | 1 | 50,000 | 50,000 | |
| 合　　　　計 | | | | | 340,000 | |

# 中文摘要


**試驗目的**

Thiazolidinediones (TZD)是一種胰島素增敏劑，用來降低第二型糖尿病患者的血糖。TZD 最明顯的副作用是會造成水份滯留，會造成全身性和週邊性的水腫，以及一些尚未確定的副作用。本研究的主要目的是在於探討 TZD 對糖尿病的眼睛病變及腎臟病變的影響。

**研究設計**

這是一個前瞻性、開放性、隨機分配以及有控制組的研究，用來探討 TZD 在糖尿病患者腎臟及眼睛所造成的影響。我們將收錄 300 個第二型的糖尿病病人，沒有明顯糖尿病的眼睛病變和腎臟病變，使用兩種口服降血糖藥物，雖然已經用到接近最大劑量，但是血糖還沒有控制的很好。 受試者納入條件為：第二型糖尿病的病友，年齡在 30 到 80 歲之間，已經使用兩種口服降血糖藥物，雖然用到接近最大劑量，糖化血色素仍然在 7%-9%之間，目前尚不考慮使用胰島素治療。 受試者排除條件為：增生性的眼底病變、腎功能不良者、肝功能不良者、肝硬化、最近 6 個月內發生過心臟衰竭、心肌缺血、目前正處於急性病的狀態、懷孕或準備要懷孕的人不適參加本研究。

我們將隨機平分成三組，分別接受 Rosiglitazone，Piolglitazone 以及

Acarbose 治療一年，然後繼續追蹤三到五年。我們將治療 6 個月來觀察短期的影響，以及治療五年來評估長期的效果。短期影響的主要研究目的為眼睛黃斑部厚度的變化，尿液白蛋白的排泄量，以及血中一些代謝性及發炎指數的改變。短期影響的次要目的將觀察空腹血糖、糖化血色素、眼底病變及腎臟功能指數的改變。長期療效的主要研究目的為眼睛出現臨床有意義的黃斑部水腫以及明顯蛋白尿。長期療效的次要目的為眼睛出現中度以上的視網膜病變，血中肌酸酐濃度加倍，腎臟衰竭，以及死亡的比例。

我們也將監測常期使用的安全議題，例如心臟衰竭、心肌梗塞、任何心臟病的發生和骨折。

**預期結果**

TZD 可以降低第二型糖尿病患者的血糖，而最明顯的副作用是會造成水份滯留。經由我們的臨床試驗，我們將可以得知 TZD 是否會造成眼睛黃斑部的水腫，改善糖尿病患者的代謝及發炎，以及減少蛋白尿的產生。我們也可以藉由長期的研究來觀察 TZD 對眼睛及腎臟的保護作用是否好處能夠大於壞處，以及長期使用的安全性。

// 英文摘要

**Objectives**

Thiazolidinediones (TZDs) are insulin sensitizers that decrease plasma glucose in type 2 diabetic patients. Thiazolidinediones can cause fluid retention and peripheral edema in diabetic patients, and the systematic fluid retention can be manifested as diabetic macular edema (DME). The overall goal of this study is to examine the effects of thiazolidinediones on the diabetic retinopathy and nephropathy.

**Study design**

This is a prospective, randomized, open-labeled, controlled design to assess the effects thiozolidinediones on the diabetic retinopathy and nephropathy. We will recruit 300 type 2 diabetic patients without significant retinopathy, nephropathy and cardiovascular disease. Inclusion criteria are type 2 diabetes, age between 30-80 years old, with microabluminuria, no significant retinopathy, on submaximal dose of sulphonylureas and metformin treatment, and A1C between 7-9%. Exclusion criteria are on insulin treatment, significant retinopathy and significant nephropathy. Patients with cardiovascular diseases, malignancy, pregnancy, in acute intercurrent illness, congestive heart failure, myocardial infarction, received PCI or CABG. All subjects will receive EKG and CXR before randomization.

These subjects will be randomized equally to 3 groups: acarbose, rosiglitazone and pioglitazone. We will follow up for 6 months to investigate the short-term effects and 5 years to evaluate the long-term outcomes. The primary study end point of short-term study will be the macular thickness changes measured by optical coherence tomography, the changes in the level of urinary albumin-to-creatinine ratio,

circulating metabolic parameters and adipocytokines during thiozolidinediones treatment. Secondary end point will be fasting blood glucose, A1C levels, development of clinically significant macular edema, serum creatinine change in patients with no history of diabetic retinopathy and nephropathy at baseline.

The primary study end point of long-term study will be the development of clinically significant macular edema and the time from the base-line visit to the first detection of overt nephropathy. Secondary end points include the development of greater than moderate NPDR, the time to the first event of the time from the base-line visit to a doubling of the serum creatinine concentration, end-stage renal disease, or death.

We also monitor the long-term safety issue, such as congestive heart failure, myocardial infarction, any cardiovascular event, and fracture.

**Expected Results**

TZDs can decrease plasma glucose in type 2 diabetic patients, but the major side effects are able to cause fluid retention. This prospective study will be able to test whether thiozolidinediones causes macular edema and to evaluate whether thiozolidinediones delays onset of diabetic retinopathy. We also will be able to find the changes in the level of urinary albumin-to-creatinine ratio, circulating metabolic parameters and adipocytokines between the treatment of TZDs and Acrbose. We can compare the time from the base-line visit to the first detection of overt nephropathy, the time to the first event of the composite end point of the time from the base-line visit to a doubling of the serum creatinine concentration, end-stage renal disease, or death.

# Thiazolidinedione 和糖尿病視網膜病變及腎病變的相關性研究

# The effects of thiazolidinedione on the diabetic retinopathy and nephropathy

## Background and Rationale

Thiazolidinediones (TZDs) are insulin sensitizers that decrease plasma glucose and improve lipid profile in type 2 diabetic patients (Yki-Jarvinen 2004). TZDs initiate their action by binding to peroxisome proliferator-activated receptor-γ (PPARγ) (Yki-Jarvinen 2004). Although PPARγ receptors are most abundant in fat cells (Yki-Jarvinen 2004, Bays 2004) they have also been demonstrated in kidney in mesangium and glomerulus (Guan 2001, Asano 2000). Studies in rodents suggest that TZDs can prevent diabetic nephropathy (Guan 2001, Ishiki 2000, McCarthy 2000) and reduction in microalbuminuria has been demonstrated in T2DM patients treated with TZDs in open-labeled trials (Iwano 1998, Bakris 2003). Inhibition of transforming growth factor-b1 and genes involved in collagen/fibronectin formation and reduced serum/renal interstitial tumor necrosis factor-α (TNF-α) levels (Moriwaki 2003, Kalantarinia 2003) have been suggested as potential mechanisms for renoprotective effect of TZDs (Guan 2001), but the precise mechanism(s) via which TZDs reduce albuminuria is yet to be defined.

Fluid retention and peripheral edema are described in 5% to 7% of patients using thiazolidinediones alone or in conjunction with other oral agents or up to 15% of patients using pioglitazone with insulin (Niemeyer 2003). Thiazolidinediones can cause fluid retention and peripheral edema in diabetic patients (Bresnick 1986, Niemeyer 2002, Mudaliar 2003), and the systematic fluid retention can be manifested as diabetic macular edema (DME) (Perkovich 1988, Tokuyama 2000). A few reports have suggested that an association between thiazolidinediones and diabetic macular edema (Colucciello 2005, Ryan 2006, Toshiyuki 2008, Fong 2009). In a recent retrospective study, a worsening of DME after thiazolidinediones (pioglitazone and/or rosiglitazone) treatment was estimated to be 1.5% and 2.6% of the cases (Ryan et al

2006). However, the investigators did not measure the visual acuity or evaluate the DME before thiazolidinediones treatment. Thus, it could not be determined whether their findings were associated with the thiazolidinediones treatment or the natural courses of diabetic retinopathy.

Adiponectin, whose secretion is enhanced by TZDs, reduces TNF-α levels, suppresses inflammatory actions of TNF-α (Goldstein 2004), enhances endothelium-dependent and independent vasodilation, stimulates nitric oxide (NO) production, and inhibits endothelial cell proliferation (Prabhakar 2004, Stehouwer 2004). These vascular effects of adiponectin could contribute to reduced albuminuria in T2DM patients treated with TZDs. Elevated plasma free fatty acid (FFA) levels (Yu 2004) and insulin resistance (Palaniappan 2003, Groop 1993) also have been associated with microalbuminuria, the precursor of diabetic nephropathy.

The previous reports had found that thiazolidinediones can reduce the microalbuminuria, but this effect may be come from better glycemic control. In this study, we use active control group to test whether this effects is independent of glycemic control. Therefore, we will be carried out to investigate the effects of thiazolidinediones on the macular edema, diabetic retinopathy, urinary albumin excretion, cardiovascular risk profiles and circulating adipocytokines in T2DM patients.

FDA recommends that healthcare professionals in FDA Drug Safety Communication: Ongoing review of Avandia (rosiglitazone) and cardiovascular safety. Safety Announcement [02-22-2010]
http://www.fda.gov/Drugs/DrugSafety/PostmarketDrugSafetyInformationforPatientsandProviders/ucm201418.htm

- Follow the recommendations in the drug label when prescribing rosiglitazone. This includes a *Boxed Warning* stating that:
    - Use of rosiglitazone in patients with established NYHA Class III or IV heart failure is contraindicated. Further, rosiglitazone is not recommended in patients with symptomatic heart failure.
    - Rosiglitazone causes or exacerbates congestive heart failure in some patients. Healthcare professionals should monitor for the signs and symptoms of heart failure (including excessive, rapid weight gain, difficulty breathing, and/or swelling) after starting treatment and after dose increases of rosiglitazone. If heart failure signs and symptoms occur, the heart failure should be managed appropriately and discontinuation or dose reduction of rosiglitazone must be considered.
    - Available data on rosiglitazone and risk of myocardial ischemia are inconclusive. A meta-analysis of 42 clinical studies (mean duration 6 months; 14,237 total patients), most of which compared rosiglitazone to placebo, found an association between rosiglitazone use and an increased risk of myocardial ischemic events such as angina or heart attack. Three other studies (mean duration 41 months; 14,067 total patients), comparing rosiglitazone to other oral diabetes medications or placebo, have not confirmed or excluded this risk. The recently completed RECORD study, currently being reviewed by FDA, is one of these three studies.
- Discuss with patients the risks of rosiglitazone treatment, taking into account the clinical utility of rosiglitazone, the risks/benefits of other antidiabetic medications, and the risks associated with poorly controlled blood glucose.
- Discuss with patients the importance of adhering to their diabetes medication regimen.
- Report any adverse events associated with the use of rosiglitazone to FDA's MedWatch program using the information at the bottom of the page.

## Overall Goal and Specific Aims

The overall goal of this study is to examine the effects of thiazolidinediones on the diabetic retinopathy and nephropathy.

The specific aims are:

1. To investigate the short-term effects of thiazolidinediones on the macular thickness measured by optical coherence tomography and the long-term effects of thiazolidinediones on the clinically significant macular edema and diabetic retinopathy documented by color photography and fluoroscein angiography.

2. Short-term effects of thiazolidinediones on the change of urine albumin excretion and serum cardiovascular risk profiles and long-term effects of thiazolidinediones on the estimated GFR change and progression to overt diabetic nephropathy.

# Experimental Design

## Study design

This is a prospective, randomized, open-labeled, controlled design to assess the effects thiozolidinediones on the diabetic nephropathy in Taipei Veterans General Hospital. We will recruit 300 type 2 diabetic subjects treated with submaximal dose of sulphonylureas and metformin, and without optimal glycemic control (a1C between 7% and 9%). These patients will be randomly assigned to add rosiglitazone (n=100), pioglitazone (n=100) or acarbose (n=100), and will expected two third of patients accepted their randomization. Patients in each treatment group are on angiotensin-converting enzyme inhibitor or angiotension receptor blocker and statins. We will intervention for one year, and follow up for 6 months to investigate the short-term effects and 5 years to evaluate the long-term outcomes.

## Subjects

Type 2 diabetic patients with microabluminuria and no significant retinopathy will be invited to participant this study. Inclusion criteria are type 2 diabetes, age between 30-80 years old, with microabluminuria, no significant retinopathy, on submaximal dose of sulphonylureas and metformin treatment, and A1C between 7-9%. Exclusion criteria are on insulin treatment, significant retinopathy (greater than moderate non-proliferative retinopathy) and significant nephropathy (overt proteinuria or serum Cr >1.50 mg/dL). Patients with cardiovascular diseases, malignancy, pregnancy, in acute intercurrent illness, congestive heart failure (CHF, according to New York heart Association, NYHA functional class III to IV), myocardial infarction, received PCI or CABG or liver cirrhosis will be also excluded. All subjects will receive EKG and

CXR before randomization.

**Procedures**

The procedures and measurements were specified in a manual of operations. The patients were examined at the time of randomization, 4, 8 and 12 weeks after randomization, every 3 months for 1 year, and then every 6 months for 5 years. A clinical examination, measurements of the blood pressure, the urinary albumin excretion, the serum creatinine concentration, and the glycosylated hemoglobin concentration and other laboratory evaluations were performed at each visit. Glomerular filtration rate was estimated using a prediction formula from the 4-variable Modification of Diet and Renal Disease study equation (Steven 2006). All assessments of urine and blood were performed at a central laboratory.

Patients continued to receive their usual care for diabetes. Arterial hypertension will be treated with a stepwise approach with the target systolic and diastolic blood pressure was less than 140/90 mmHg. All patients will be initially prescribed an ACE inhibitor or an angiotensin II–receptor antagonist. If a patient has hypertension, thiazides, calcium-channel blockers, and beta-blockers will be added as needed. Isolated instances of raised fasting serum cholesterol concentrations or combined dyslipidemia were treated with statins (atorvastatin, with a maximum of 40 mg daily, or the equivalent). Aspirin (100 mg per day) will be given as secondary prevention to patients with a history of ischemic cardiovascular disease. No restriction on dietary salt or protein was implemented.

**Eye examinations**

Ocular examinations included recordings of best-corrected visual acuity, intraocular

pressure (non-contact tonometry), slit-lamp biomicroscopy, and direct and silt-lamp indirect ophthalmoscopy (pharmacological mydriasis). Color photographs of the retinas were also obtained. Macula- and disc-centered views were taken at an angle of $50^{o}$ with a fundus camera after pharmacological mydriasis. We used the alternative classification of the Wisconsin Epidemiological Study of Diabetic Retinopathy and the Standard Photographs (SP) 2A, 3 and 8A to classify the retinopathy. This classification is based on all fields together and provides an overall retinopathy scale.

- Level 10: no retinopathy.
- Level 21: microaneurysms only or blot hemorrhages or soft exudates in the absence of microaneurysms.
- Level 31: microaneurysms and one or more of the following: retinal hemorrhages, but total of hemorrhages and microaneurysms (H/Ma) less than standard photograph (SP) 2A; hard exudates (HE) less than SP3; soft exudates (SE) questionably present; intraretinal microvascular abnormalities (IRMA) questionably present; venous beading (VB) questionably present; or small venous loops definitely present.
- Level 41: microaneurysms and one or more of the following, but definition of level 51 not met: H/Ma greater than or equal to SP2A; HE greater than or equal to SP3; SE definitely present; IRMA definitely present; VB definitely present; or large venous loops or reduplication definitely present.
- Level 51: microaneurysms and one or more of the following :IRMA definitely present and greater than or equal to SP8A ; H/Ma greater than or equal to SP2A in one field; SE definitely present in two or more fields; VB definitely present in two or more fields.
- Level 60+: characterized by all levels of PDR.

***Optical Coherence Tomography***

One new technique is optical coherence tomography, which projects a pair of near-infrared beams from a diode through the pupil of the eye and then through the vitreous, retina, and choroid. The structures of the eye disrupt the coherence of the two beams, producing an interference pattern detected by the measuring system of the instrument and dependent on the optical reflectance and anatomical thickness of the retinal structures (Huang 1991). In the most commonly used protocol, the instrument produces a series of six radially oriented scans at equal intervals around a circumference of 360 degrees. The scans pass through the fixation point of the patient's eye (the center of the fovea). Each scan makes multiple measurements of retinal thickness; the most recent version of the instrument makes up to 768 measurements. The images produced appear to be good approximations of the cross-sectional anatomy of the retina.

Measurements of mean retinal thickness along these radii are plotted, along with a pseudocolor map of retinal thickness that enhances the visual interpretability of the images. Optical coherence tomography can therefore be used for the evaluation and follow-up of patients with diabetic macular edema (Rivellese 2000). Because the method requires the projection of light onto the retina, it is subject to error in the presence of interfering opacities such as cataracts, corneal opacities, or vitreous hemorrhage. However, day-to-day variation in the same patient appears to be small (Massin 2001). In diabetic macular edema, optical coherence tomography can provide objective, quantitative measurements that are not possible with other methods. An advanced version of this device, involving a different optical system and a titanium–aluminum oxide laser, provides even more striking images, which display the cellular anatomy of the retinal layers in nearly the detail of a histologic section (Drexler 1986).

## Assays

The $HbA_{1c}$ was measured using high-performance liquid chromatography (HPLC) instruments (HLC-723G7, Tosoh, Japan) with a reference range of 4.2% – 5.8%. The inter-assay with between-batch coefficient of variance (CV) was less than 2.0% at mean $HbA_{1c}$ levels between 4.4% and 8.2%. The urinary albumin concentration was determined by nephelometry and the serum creatinine concentration by Jaffe reaction with the use of a Hoffmann–LaRoche kit. Glomerular filtration rate was estimated using a prediction formula from the 4-variable Modification of Diet and Renal Disease study equation (Stevens 2006).

Plasma glucose, fasting and pooled postprandial triglycerides, lipoproteins, and free fatty acids were measured using standard laboratory techniques in the core laboratory of the Einstein General Clinical Research Center. High sensitivity-C-reactive protein (hsCRP) was measured by latex-enhanced turbitimetric assay (CRP Ultra Wide Range Reagent Kit; Equal Diagnostics, Exton, PA). glycosylated hemoglobin (HbA1c) was assayed by HPLC; serum creatinine and other clinical tests were performed using standard techniques. PAI-1 antigen was measured using Lincoplex human serum adipokine panel (Linco Research, St. Charles, MO), adiponectin by RIA (Linco), and insulin by RIA in the Einstein Diabetes Research and Training Center Hormone ssay Core.

## Outcome Measures

### *Short-term study (3-6 months)*

The primary study end point will be the macular thickness changes during thiozolidinediones treatment measured by optical coherence tomography for

retinopathy, and The changes in the level of urinary albumin-to-creatinine ratio, circulating metabolic parameters and adipocytokines. Secondary end point will be the development of clinically significant macular edema, fasting blood glucose, and A1C.

***Long-term study (3-5 years)***

The primary study end point will be the development of clinically significant macular edema for retinopathy, and the time from the base-line visit to the first detection of overt nephropathy, defined by a urinary albumin excretion rate in an overnight specimen that was greater than 300 mg/g Cr and at least 30 percent higher than the base-line rate on at least two consecutive visits. Secondary end points will be A1C level, development of greater than moderate NPDR, the first event of the composite end point of the time from the base-line visit to a doubling of the serum creatinine concentration, end-stage renal disease, or death. The doubling of the serum creatinine concentration was defined as the first serum creatinine value that was twice the base-line value, as confirmed by a second serum creatinine value obtained at least four weeks after the initial doubling. End-stage renal disease was defined by the need for long-term dialysis or renal transplantation.

***Long-term safety issue (3-5 years)***

The main adverse effects reported with thiazolidinediones are weight gain, pedal edema, bone loss and precipitation of congestive heart failure in at-risk individuals, without any increase in CVD/all-cause mortality. Rosiglitazone has been reported in meta-analyses to be associated with possible increased risk of cardiovascular disease (CVD) events. Pioglitazone appears to be associated with decreased CVD events in most studies/meta-analyses. Long-term exposure of type 2 diabetic patients to thiazolidinediones was associated with higher odds of fractures among women without a significant increase in odds of fractures among men.

We will monitor for the signs and symptoms of heart failure (including excessive, rapid weight gain, difficulty breathing, and/or swelling), myocardial ischemia, all cardiovascular event, and fracture.

**Statistical analysis**

The statistical analysis was performed with SPSS for Windows version 17.0 (SPSS, Inc., Chicago, IL). Values before and after treatment within each group were analyzed using paired Student's t-test. Comparison between groups was performed using analysis of variance with Bonferroni/Dunn post hoc testing. Pearson's correlations between continuous variables were used as a measure of association. Stepwise multiple linear regression analysis was performed to examine multiple correlations among variables. Data are presented as mean ±S.E. P-value <0.05 was considered statistically significant.

Comparisons of means between the thiazolidinediones and control groups were performed using a 2-sample *t*-test for populations with normal distribution. Categorical data expressed in percentages were compared using a $\chi^2$ test. When the expected frequencies were excessively small, Fisher exact tests were performed. Analysis of ophthalmic data excluded information from patients who underwent only 1 ophthalmic evaluation during the study period. Survival analysis using the Kaplan-Meier method was used to compare data on cumulative rates with various follow-up times and eyes as a unit. Differences between survival curves of the 2 groups were assessed with both Wilcoxon and log-rank tests. Cumulative percentages were obtained from survival curves at 6 months and annually for up to 5 years of follow-up. $P \leq 0.05$ was defined as statistically significant.

## Expected Results

***Short-term study (3-6 months)***

The two clinical objectives of the current study will be able to identify patients who were noted to have both macular edema documented by OCT and clinically significant macular edema during thiozolidinediones use and to evaluate whether thiozolidinediones use can delay onset of proliferative diabetic retinopathy (PDR).

We will find the changes in the level of urinary albumin-to-creatinine ratio, circulating metabolic parameters and adipocytokines between the treatment of TZDs and Acrbose.

***Long-term study (3-5 years)***

This prospective study will be able to test whether thiozolidinediones causes macular edema and to evaluate whether thiozolidinediones delays onset of proliferative diabetic retinopathy (PDR).

We can compare the time from the base-line visit to the first detection of overt nephropathy between TZDs and Acarbose treatment. We also can investigate the time to the first event of the composite end point of the time from the base-line visit to a doubling of the serum creatinine concentration, end-stage renal disease, or death.

## References


- Aronoff S, Rosenblatt S, Braithwaite S, et al.. Pioglitazone hydrochloride monotherapy improves glycemic control in the treatment of patients with type 2 diabetes: a 6-month randomized placebo-controlled dose-response study. The Pioglitazone 001 Study Group. Diabetes Care 2000; 23:1605–11.
- Asano T, Wakisaka M, Yoshinari M et al. Peroxisome proliferator-activated receptor gamma1 (PPARgamma1) expresses in rat mesangial cells and PPARgamma agonists modulate its differentiation. Biochim Biophys Acta 2000; 1497: 148–154.
- Bakris G, Viberti G, Weston WM et al. Rosglitazone reduces urinary albumin excretion in type II diabetes. J Hum Hypertens 2003; 17: 7–12.
- Bays H, Mandarino L, DeFronzo RA. Role of the adipocytes FFA and ectopic fat in the pathogenesis of type 2 diabetes mellitus. PPAR agonists provide a rational therapeutic approach. J Clin Endocrinol Metab 2004; 89: 463–478.
- Bresnick GH. Diabetic macular edema. A review. Ophthalmology 1986; 93: 989–997.
- Chun SH, Li AH. Arch Ophthalmol. Association of proliferative diabetic retinopathy with insulin use and microalbuminuria. 2010 Jan;128(1):146; author reply 146-7. No abstract available.
- Colucciello M. Vision loss due to macular edema induced by rosiglitazone treatment of diabetes mellitus. Arch Ophthalmol 2005; 123:1273–5.
- Fong DS, Contreras R. Am J Glitazone use associated with diabetic macular edema. Ophthalmol. 2009 Apr;147(4):583-586.
- Goldstein BJ, Scalia R. Adiponectin: a novel adipokine linking adipocytes and vascular function. J Clin Endocrinol Metab 2004; 89: 2563–2568.
- Groop L, Ekstrand A, Forsblom C et al. Insulin resistance, hypertension and microalbuminuria in patients with type 2 (non-insulin-dependent) diabetes mellitus. Diabetologia 1993; 36: 642–647.
- Guan Y, Breyer M. Peroxisome proliferator-activated receptors (PPARs). Novel

therapeutic targets in renal disease. Kidney Int 2001; 60: 14–30.

- Herz M, Johns D, Reviriego J, et al.. A randomized, double-blind, placebo-controlled, clinical trial of the effects of pioglitazone on glycemic control and dyslipidemia in oral antihyperglycemic medication-naive patients with type 2 diabetes mellitus. Clin Ther 2003; 25:1074–95.
- Ishiki K, Haneda M, Koya D et al. Thiazolidinedione compounds ameliorate glomerular dysfunction independent of their insulinsensitizing action in diabetic rats. Diabetes 2000; 49: 1022–1032.
- Iwano E, Kanda T, Nakatani Y et al. Effect of troglitazone on microalbuminuria in patients with incipient diabetic nephropathy. Diabetes Care 1998; 21: 2135–2139.
- Kalantarinia K, Awad AS, Siragy H. Urinary and renal interstitial concentrations of TNF-a increase prior to albuminuria in diabetic rats. Kidney Int 2003; 64: 1208–1213.
- Lambley RG, Vahdani K, Konstantinidis A, Booth A.Rosiglitazone and pioglitazone. Beware macular oedema. BMJ. 2009 Sep 22;339:b3856. doi: 10.1136/bmj.b3856. No abstract available. Erratum in: BMJ. 2009;339. doi: 10.1136/bmj.b4044.
- Liazos E, Broadbent DM, Beare N, Kumar N.Spontaneous resolution of diabetic macular oedema after discontinuation of thiazolidenediones. Diabet Med. 2008 Jul;25(7):860-2.
- Lincoff AM, Wolski K, Nicholls SJ, et al.. Pioglitazone and risk of cardiovascular events in patients with type 2 diabetes mellitus: a metaanalysis of randomized trials. JAMA 2007; 298:1180–8.
- McCarthy KJ, Routh RE, Shaw W et al. Troglitazone halts diabetic glomerulosclerosis by blockade of mesangial expansion. Kidney Int 2000; 58: 2341–2350.
- Moriwaki Y, Yamamoto T, Shibutani Y et al. Elevated levels of interleukin-18 and tumor necrosis factor-a in serum of patients with type 2 diabetes mellitus: relationship with diabetic nephropathy. Metabolism 2003; 52: 605–608.
- Mudaliar S, Chang AR, Henry RR. Thiazolidinediones, peripheral edema, and type

2 diabetes: incidence, pathophysiology, and clinical implications. Endocr Pract 2003; 9:406–16.

- Niemeyer NV, Janney LM. Thiazolidinedione-induced edema. Pharmacotherapy 2002;22:924–929.
- Oshitari T, Asaumi N, Watanabe M, Kumagai K, Mitamura Y. Severe macular edema induced by pioglitazone in a patient with diabetic retinopathy: a case study. Vasc Health Risk Manag. 2008;4(5):1137-40.
- Palaniappan L, Carnethon M, Fortmann SP. Association between microalbuminuria and the metabolic syndrome: NHANES III. Am J Hypertens 2003; 16: 952–958.
- Perkovich BT, Meyers SM. Systemic factors affecting diabetic macular edema. Am J Ophthalmol 1988; 105: 211–212.
- Prabhakar S. Role of nitric oxide in diabetic nephropathy. Sem Nephrol 2004; 24: 333–344.
- Ryan EH Jr, Han DP, Ramsay RC, Cantrill HL, Bennett SR, Dev S, Williams DF. Diabetic macular edema associated with glitazone use. Retina. 2006; 26: 562-70.
- Shen LQ, Child A, Weber GM, Folkman J, Aiello LP. Rosiglitazone and delayed onset of proliferative diabetic retinopathy. Arch Ophthalmol. 2008 Jun;126(6):793-9.
- Sivagnanam G. Rosiglitazone and macular edema. CMAJ. 2006 Aug 1;175(3):276.
- Stehouwer CDA, Henry RMA, Dekker JM et al. Microalbuminuria is associated with impaired brachial artery, flow-mediated vasodilation in elderly individuals without and with diabetes: further evidence for a link between microalbuminuria and endothelial dysfunction. Kidney Int 2004; 66: S42–S44.
- Stevens LA, Coresh J, Greene T, Levey AS. Assessing kidney function--measured and estimated glomerular filtration rate. *N Engl J Med.* 2006; 354(23): 2473-2483.
- Stumvoll M, Hfaring HU. Glitazones: clinical effects and molecular mechanisms. Ann Med 2002;34:217–224.
- Tatti P, Arrigoni F, Longobardi A, Costanza F, Di Blasi P, Merante D.

Retrospective analysis of rosiglitazone and macular oedema in patients with type 2 diabetes mellitus. Clin Drug Investig. 2008;28(5):327-32.

- Tokuyama T, Ikeda T, Sato K. Effects of haemodialysis on diabetic macular leakage. Br J Ophthalmol 2000; 84: 1397–1400.
- Wang CH, Weisel RD, Liu PP, Fedak PW, Verma S. Glitazones and heart failure: critical appraisal for the clinician. Circulation 2003;107: 1350.
- Yki-Jarvinen H. Thiazolidinediones. N Engl J Med 2004; 351: 1106–1118.
- Yu Y, Suo L, Yu H et al. Insulin resistance and endothelial dysfunction in type 2 diabetic patients with or without microalbuminuria. Diabetes Res Clin Pract 2004; 65: 95–104.

## 十三、近三年內執行及申請中之研究計畫

| 計畫名稱及編號 | 計畫內擔任之工作 | 起迄年月 | 補助或委託機構 | 申請(執行)情形 |
|---|---|---|---|---|
| 第一型類胰島素生長因子和生長激素在肢端肥大症患者存活率所扮演的角色(V97C1-171) | 主持人 | 2008/1/1 ~ 2009/1/1 | 台北榮總 | 已結案 |
| 第一型類胰島素生長因子在肢端肥大症患者眼底變化及心臟功能所扮演的角色(V98C1-154) | 主持人 | 2009/1/1 ~ 2010/1/1 | 台北榮總 | 已結案 |
| 第一型類胰島素生長因子跟第二型糖尿病老人發生心臟衰竭的關係(V99C1-158) | 主持人 | 2010/1/1 ~ 2011/1/1 | 台北榮總 | 執行中 |
| 短期間胰島素的治療可以改善剛發病第二型糖尿病病人 | 協同主持人 | 2010/8/1 ~ 2012/7/31 | 國科會 | 申請/審查中 |
| 80 歲以上生活可以自理的第二型糖尿病患者， | 主持人 | 2010/8/1 ~ 2013/7/31 | 國科會 | 申請/審查中 |

# 研究人員近五年內研究成果統計及獲獎勵情形（表 A）

1. 五年內曾懷孕生產或請育嬰假者，得填寫近七年內研究成果統計及獲獎情形，但須附證明文件。
2. 五年內曾服國民義務役者，得加上實際服役時間延長選填研究成果及獲獎情形，但須附證明文件。

(修正：2009/11/18)

| | |
|---|---|
| 研究人員姓名 | 陳涵栩; Chen, Harn-Shen |
| 任職機關系所 | 國立陽明大學 內科<br>台北榮民總醫院 內科部 內分泌暨新陳代謝科 |

※本表所填寫下列各項數量資料均應以研究人員個人資料表所填列之資料為依據。

(一)請填寫五年內(2005.1.1 迄今)已發表或已被接受發表之研究論文數量

| 研究成果 / 作者序 | SCI、SSCI、EI 期刊論文（包括填表說明六(一)之 1.所列四類論文：正式論文、簡報型論文、病例報告、綜合評論） | | | | 其他學術期刊論文(左列 4 類以外之期刊論文) |
|---|---|---|---|---|---|
| | SCI 論文 | SSCI 論文 | EI 論文 | 加重計分之國內非 SCI 優良期刊論文(發表於填表說明之附表 2 所列之期刊) | |
| 第一作者論文篇數 | 8 | | | 1 | |
| 非第一作者之通訊作者論文篇數 | | | | | |
| 非第一或通訊作者之其他序位作者論文篇數 | 1 | | | 1 | |
| **總篇(件)數**（以上三項總和） | 9 | 0 | 0 | 2 | |

SCI 、SSCI、EI 之期刊論文資料，可就近至各大學圖書館、國科會科技政策研究與資訊中心等查閱或上網檢索。上述 SCI、SSCI 及 EI 期刊資料以 2007 年版本為準。

(二)請填寫五年內(2005.1.1 迄今)已獲得或已刊登之下列研究成果數量

| 成果名稱 | 專利 | 技轉 | 研討會論文摘要- | 專書或專書章節 | 其他 |
|---|---|---|---|---|---|
| （件、冊、章、篇）數 | | | | | |

(三)請填寫五年內(2005.1.1 迄今)獲得獎勵情形

| 年 度 | 請選填下列獎項名稱：傑出獎、吳大猷獎、其他獎(請填獲獎名稱) |
|---|---|
| 1. 94 年度(94.1.1~94.12.31) | 默沙東優秀論文獎(中華民國內分泌學會) |
| 2. 95 年度(94.1.1~95.12.31) | 衛生署獎勵醫療機構之醫事人員從事臨床研究 |
| 3. 96 年度(96.1.1~96.12.31) | 衛生署獎勵醫療機構之醫事人員從事臨床研究 |
| 4. 98 年度(98.1.1~迄今) | 輝瑞優秀論文獎(中華民國內分泌學會) |

# 研究人員近五年內研究表現指數（RPI）統計（表 B）

(修正：2009/11/18)

| | |
|---|---|
| 姓　　名： | 陳涵栩;　Chen, Harn-Shen |
| 機關系所： | 國立陽明大學 醫學院內科 及台北榮民總醫院 內科 |
| 研究年資：（請打✓） | ☑滿 5 年以上(選最佳 10 篇)； ☐滿 4 年(選最佳 7 篇)； ☐滿 3 年(選最佳 5 篇)； ☐未滿 3 年(選最佳 3 篇)<br>1. 五年內曾懷孕生產或請育嬰假者，得於七年內選擇上列所須論文篇數，研究年資則扣除 2 年後，於上列四項年資中勾選年資，並請附上懷孕生產或請育嬰假證明文件。<br>2. 五年內曾服國民義務役者，得加上實際服役時間延長選擇上列所須論文篇數，研究年資則為扣除實際服役時間，於上列四項年資中勾選您的年資，並請附上服國民義務役證明文件。 |

| 序號 | 成果類別代碼（參看填表說明之三） | 五年內(2005.1.1 迄今)代表性研究成果名稱<br>★1. 學術論文必須填寫所有作者(按期刊所刊登之原排序)、著作名稱、期刊名稱、年份、卷期、起迄頁數。<br>★2. 專利必須填寫專利名稱、發明人、證書號碼、國別、專利期限。<br>★3. 技術移轉必須填寫技術名稱、技轉金額及對象、年份。<br>★4. 刊登雜誌分類排名以 2008 年版本之 SCI 及 SSCI 資料為準。 | 論文性質分數（C） | 刊登雜誌分類分數（J） | 作者排名分數（A） | 分數（CxJxA） |
|---|---|---|---|---|---|---|
| 1 | 01 | **Harn-Shen Chen**, Tzu-En Wu, Hong-Da Lin, Tjin-Shing Jap, Li-Chuan Hsiao, Shen-Hung Lee, Shu-Hsia Lin. Hemoglobin A1C and Fructosamine for Assessing Glycemic Control in Diabetic Patients with CKD Stages 3 and 4. **American Journal of Kidney Diseases** 2010: 55: 867-874 (排名=10%) | 3 | 5 | 5 | 75 |
| 2 | 01 | **Harn-Shen Chen**, Tzu-En Wu, Chi-Chang Juan, and Hong-Da Lin. Myocardial heat shock protein 60 expression in the insulin resistant and diabetic rats. **Journal of Endocrinology** 2009; 200: 151-157 (排名=50%) | 3 | 3 | 5 | 45 |
| 3 | 01 | **Harn-Shen Chen**, Tzu-En Wu, Tjin-Shing Jap, Li-Chuan Hsiao, Shen-Hung Lee, Hong-Da Lin. Beneficial effects of insulin on glycemic control and ß-cell function in newly diagnosed type 2 diabetes with severe hyperglycemia after short-term intensive insulin therapy. **Diabetes Care** (Impact Factor 7.349) 2008; 31: 1927-1932 | 3 | 7.349 | 5 | 110.24 |
| 4 | 01 | **Harn-Shen Chen**, Tzu-En Wu, Tjin-Shing Jap, Ru-Lin Chen, Hong-Da Lin. Effects of Health Education on Glycemic Control during Holiday Time in Type 2 Diabetic Patients. **The American Journal of Managed Care** 2008; 14: 41-47. (排名=28%) | 3 | 4 | 5 | 60 |
| 5 | 01 | **Harn-Shen Chen**, Tzu-En Wu, Tjin-Shing Jap, Shu-Hsia Lin, Li-Chuan Hsiao, Hong-Da Lin. Improvement of glycemic control in subjects with type 2 diabetes by self-monitoring of blood glucose: Comparison of two management programs adjusting bedtime insulin dosage. **Diabetes, Obesity and Metabolism** 2008; 10: 34-40. (排名=24%) | 3 | 4 | 5 | 60 |

| | | | | | | |
|---|---|---|---|---|---|---|
| 6 | 01 | **<u>Harn-Shen Chen</u>**, Tzu-En J. Wu, Tjin-Shing Jap, Ron-A Lu, Mei-Li Wang, Ru-Lin Chen, Hong-Da Lin. Subclinical hypothyroidism is a risk factor for nephropathy and cardiovascular diseases in type 2 diabetic patients. **Diabetic Medicine** 2007; 24: 1336-1344. (排名=41%) | 3 | 3 | 5 | 45 |
| 7 | 01 | **<u>Harn-Shen Chen</u>**, Jia Jia, Hou-fen Su, Hong-Da Lin, Jaw-Wen Chen, Shing-Jong Lin, Jia-Ying Yang, Hui-Chin Lai, Ruben Mestril, Ping H Wang. Down-regulation of the constitutively expressed Hsc70 in diabetic myocardium is mediated by insulin deficiency. **Journal of Endocrinology** 2006; 190: 435-442. (排名=50%) | 3 | 3 | 5 | 45 |
| 8 | 01 | **<u>Harn-Shen Chen</u>**, Tzu-En Wu, Tjin-Shing Jap, Shen-Hung Lee, Mei-Li Wang, Ron-A Lu, Ru-Lin Chen, Hong-Da Lin. Decrease heart rate variability but preserve the postural blood pressure change in type 2 diabetes with microalbuminuria. **Journal of the Chinese Medical Association** 2006; 69: 254-258. | 3 | 1 | 5 | 15 |
| 9 | 01 | **<u>Harn-Shen Chen</u>**, Yue-Xin Shan, Tung-Lin Yang, Hong-Da Lin, Jaw-Wen Chen, Shing-Jong Lin, Ping H. Wang. Insulin deficiency down-regulated Hsp60 and IGF-1 Receptor signaling in diabetic myocardium. **Diabetes** 2005; 54:175-181. (IF=8.398) | 3 | 8.398 | 5 | 126.0 |
| 10 | 01 | Jap TS, Chiu CY, Won JG, Wu YC, **<u>Chen HS</u>**. Novel mutations in the MEN1 gene in subjects with multiple endocrine neoplasia-1. **Clinical Endocrinology (Oxf)**. 2005; 62: 336-42. (排名=36%) | 3 | 4 | 0.5 | 6 |
| 積分（以上各項研究成果分數之總和） | | | | | | 587.24 |
| 研究表現指數(RPI)【(積分 x100)/ 指標上限滿分】 | | | | | | 78.30 |

★註 1. 已被接受但未出刊之論文須附接受函或相關證明文件，技術移轉須附上合約書，專利須附上專利證書，採相同貢獻作者計分者須附該論文註明「相同貢獻作者」部份之電子檔，前述文件請掃描附於本表之後一併上傳，未附者將不採計。

★註 2. 申請人填寫本表之資料經核對結果，若填寫不實將予更正，無法辨識者將取消計分；蓄意造假者，其申請案不予通過外，並送本會學術倫理審議委員會按情節輕重程度議處。

行政院國軍退除役官兵輔導委員會台北榮民總醫院
TAIPEI VETERANS GENERAL HOSPITAL, VAC
201 SHIH-PAI ROAD, SEC. 2
TAIPEI, TAIWAN 11217
REPUBLIC OF CHINA
TEL: (886)-2-2871-2121(30 LINES)

# 同 意 臨 床 試 驗 證 明 書

查本院新陳代謝科陳涵栩醫師所提研究計畫：「Thiazolidinedione 和糖尿病視網膜病變及腎病變的相關性研究」（本院編號：201004014IA）臨床試驗案，已於九十九年六月七日經本院人體試驗委員會(一)第 5 次會議審查通過，特此證明。 有效期限至一 OO 年六月六日

台北榮民總醫院
人體試驗委員會
主任委員
蘇東平

中 華 民 國 九 十 九 年 六 月 二 十 二 日

Jun 22, 2010

To Whom It May Concern:

RE: The effects of thiazolidinedione on the diabetic retinopathy and nephropathy.

Principle Investigator: Harn-Shen Chen, M.D.
Protocol Version: 2010/6/21
Informed Consent Form: Version 1, Date: 2010/6/21
VGHIRB No.: 201004014IA

Above study was approved by the Institutional Review Board of the Taipei Veterans General Hospital and effective till Jun 06, 2011. The Institutional Review Board performs its functions according to written operating procedures and complies with GCP and with the applicable regulatory requirements.

Tung-Ping Su

Tung-Ping Su,M.D.
Chairman
Institutional Review Board
Taipei Veterans General Hospital
Taiwan, R.O.C.

行政院國軍退除役官兵輔導委員會台北榮民總醫院 人體試驗委員會 Institutional Review Board V. G. H., TAIPEI, VAC

人體試驗委員會
主任委員蘇東平
