# Supplementary material for: Renal Outcomes of Pioglitazone Compared with Acarbose in Diabetic Patients: A Randomized Controlled Study
Source: PLoS One. 2016 Nov 3;11(11):e0165750. doi: 10.1371/journal.pone.0165750 (PMC5094682; doi:10.1371/journal.pone.0165750)
Supplement: S2 File — (PDF) [file pone.0165750.s002.pdf]

行政院國軍退除役官兵輔導委員會台北榮民總醫院
TAIPEI VETERANS GENERAL HOSPITAL, VAC
201 SHIH-PAI ROAD, SEC. 2
TAIPEI, TAIWAN 11217
REPUBLIC OF CHINA
TEL: (886)-2-2871-2121(30 LINES)

同 意 臨 床 試 驗 證 明 書

查本院新陳代謝科陳涵栩醫師所提研究計畫：「Thiazolidinedione 和糖尿病視網膜病變及腎病變的相關性研究」（本院編號：201004014IA）臨床試驗案，已於九十九年六月七日經本院人體試驗委員會(一)第 5 次會議審查通過，特此證明。 有效期限至一 OO 年六月六日

NCT 01175486

台北榮民總醫院
人體試驗委員會
主任委員
蘇東平

中　華　民　國　九　十　九　年　六　月　二　十　二　日

Jun 22, 2010

To Whom It May Concern:

RE: The effects of thiazolidinedione on the diabetic retinopathy and nephropathy.

Principle Investigator: Harn-Shen Chen, M.D.
Protocol Version: 2010/6/21
Informed Consent Form: Version 1, Date: 2010/6/21
VGHIRB No.: 201004014IA

Above study was approved by the Institutional Review Board of the Taipei Veterans General Hospital and effective till Jun 06, 2011. The Institutional Review Board performs its functions according to written operating procedures and complies with GCP and with the applicable regulatory requirements.

Tung-Ping Su

Tung-Ping Su,M.D.
Chairman
Institutional Review Board
Taipei Veterans General Hospital
Taiwan, R.O.C.

行政院國軍退除役官兵輔導委員會台北榮民總醫院
人體試驗委員會
Institutional Review Board
V. G. H., TAIPEI, ROC

人體試驗委員會
主任委員蘇東平

行政院國軍退除役官兵輔導委員會台北榮民總醫院
TAIPEI VETERANS GENERAL HOSPITAL, VAC
201 SHIH-PAI ROAD, SEC. 2
TAIPEI, TAIWAN 11217
REPUBLIC OF CHINA
TEL: (886)-2-2871-2121(30 LINES)

同 意 臨 床 試 驗 證 明 書

查本院新陳代謝科陳涵栩醫師所提研究計畫：「Thiazolidinedione 和糖尿病視網膜病變及腎病變的相關性研究」（本院編號：201004014IA#1）臨床試驗變更案，業經本院人體試驗委員會(一)審查通過，特此證明。 有效期限至一OO 年六月六日

Remove Rosiglitazone group

台北榮民總醫院
人體試驗委員會
主任委員
蘇東平

中 華 民 國 九 十 九 年 十 一 月 十 九 日

Nov 19, 2010

To Whom It May Concern:

RE: The effects of thiazolidinedione on the diabetic retinopathy and nephropathy.

Principle Investigator: Harn-Shen Chen, M.D.
Protocol Version: 2010/10/21
Informed Consent Form: Version 1, Date: 2010/10/21
VGHIRB No.: 201004014IA#1

Above study was approved by the Institutional Review Board of the Taipei Veterans General Hospital and effective till Jun 06, 2011. The Institutional Review Board performs its functions according to written operating procedures and complies with GCP and with the applicable regulatory requirements.

Tung-Ping Su

Tung-Ping Su,M.D.
Chairman
Institutional Review Board
Taipei Veterans General Hospital
Taiwan, R.O.C.

行政院國軍退除役官兵輔導委員會台北榮民總醫院 人體試驗委員會 Institutional Review Board V. G. H., TAIPEI, VAC

人體試驗委員會 主任委員蘇東平

正本

分類號：
保存年限：
總頁數：

行政院國軍退除役官兵輔導委員會臺北榮民總醫院　書函

地址：台北市石牌路二段 201 號
聯 絡 人：陳 亦 筑
聯絡電話：（02）2875-7384 分機：253
傳　　真：（02）2871-3241

受文者：新陳代謝科陳涵栩醫師

發文日期：中華民國 101 年 7 月 10 日
發文字號：北總教字第 1010017450 號
速別：普通件
密等及解密條件或保密期限：普通
附件：

主旨：同意台端繼續進行所提研究計畫：「Thiazolidinedione 和糖尿病視網膜病變及腎病變的相關性研究」（本院 IRB 編號：201004014IA）臨床試驗如說明，請 查照。

說明：

一、本案業經本院人體試驗委員會(一)審查同意繼續進行，有效期限至 102 年 6 月 6 日。

二、Protocol title：The effects of thiazolidinedione on the diabetic retinopathy and nephropathy.，VGHTPE-IRB No.：201004014IA。

三、TPVGH-IRB(1) had reviewed and agreed to continue approving this trial and the approval date is extented to Jun 6, 2013。

四、已核准之廣告紙本須經本院人體試驗委員會蓋戳印方可張貼。

五、依人體試驗管理辦法第 15 條規定「醫療機構於人體試驗期間，不得對外發表成果或為宣傳」。

六、若需展延研究期限，請於有效期限前 6 星期檢送計畫展延申請書至本院人體試驗委員會審查，以利展延研究期限。

七、試驗結束 3 個月內，請依規定向本院人體試驗委員會辦理結案，繳交結案報告。

八、若未於有效期限後 3 個月內提出展延或結案申請者，此計畫將於同意臨床試驗證明書之有效日之截止日後 6 個月內逕行結案。凡未依規定繳交報告或前述逕行結案之計畫主

持人，本院人體試驗委員會將於爾後6個月內不受理其新計畫之申請。

九、計畫試驗中止或終止時，應說明原因並繳交試驗結果。

正本：本院新陳代謝科陳涵栩醫師
副本：本院人體試驗委員會

行政院國軍退除役官兵輔導委員會台北榮民總醫院
